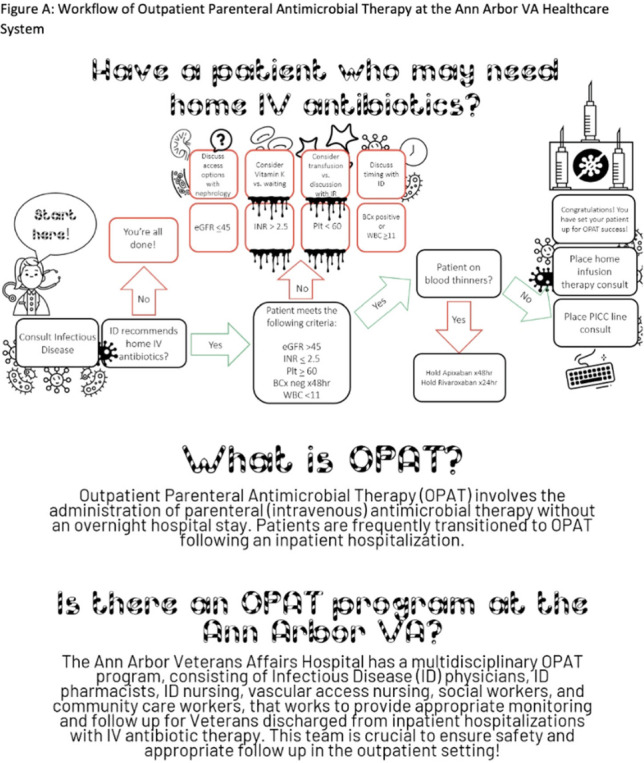# My patient needs home IV antibiotics–Now what? Assessing OPAT involvement at a Veterans’ Affairs hospital

**DOI:** 10.1017/ash.2022.168

**Published:** 2022-05-16

**Authors:** Maddy Breeden, Elizabeth Scruggs, Payal Patel, Sarah Krein, Ronald Kendall, Andrea Starnes, Tracy Lopus

## Abstract

**Background:** Outpatient parenteral antimicrobial therapy (OPAT) involves the administration of intravenous antimicrobial therapy outside the hospital. The literature suggests that inpatient providers are often unaware of OPAT programs and may not engage this multidisciplinary group in a timely fashion, leading to potentially inappropriate OPAT use. However, few studies have directly addressed this issue. We characterized current practices for coordinating OPAT and assessed provider understanding of OPAT services. We also conducted an exploratory analysis of placement of a peripherally inserted central catheter (PICC) consultation prior to an infectious disease (ID) consultation as a proxy for potentially avoidable OPAT use. **Methods:** This study was conducted between September and December 2021 at the Ann Arbor VA Healthcare System. All charts (n = 212) in which a consultation for a PICC was placed between January and September 2021 were reviewed, including free-text data entered by patient teams and inpatient progress notes in the days leading up to and following PICC consultation. Additionally, inpatient providers were surveyed using an online format regarding knowledge, utilization, and perceptions of OPAT. **Results:** Of the 212 charts reviewed, 108 patient encounters resulted in PICC placement; 80 (74.1%) were placed for the indication of home IV antibiotics. Of these, 3 (4.0%) had the PICC consult placed prior to the ID consultation. Of the 104 PICC consultations that were cancelled, 9 (8.7%) were cancelled because the ID staff did not recommend home IV antibiotics. Other reasons for cancellation included alternative device placement, duplicate order, referral to interventional radiology, failure to meet criteria, or unsuccessful placement. Of the 285 inpatient providers sent the electronic survey, 121 (46.9%) completed at least some portion. Overall, 17 respondents (14.0%) were familiar with the acronym OPAT; however, only 10 were able to expand the acronym correctly. Of the 118 respondents asked about their familiarity with the OPAT program at the local institution, 98 (83.1%) were not familiar at all or were only slightly familiar with the program. In contrast, 7 respondents (6.0%) were very or extremely familiar with the OPAT program. **Conclusions:** Further education and structural interventions are necessary to improve inpatient providers’ awareness and early engagement of local OPAT programs to ensure appropriate OPAT use. An educational intervention with an informative flowchart diagramming the steps for engaging the OPAT team could raise awareness and improve engagement when potential OPAT needs are identified (Fig. 1).

**Funding:** None

**Disclosures:** None

**Figure:**